# Genome and metagenome analyses reveal adaptive evolution of the host and interaction with the gut microbiota in the goose

**DOI:** 10.1038/srep32961

**Published:** 2016-09-09

**Authors:** Guangliang Gao, Xianzhi Zhao, Qin Li, Chuan He, Wenjing Zhao, Shuyun Liu, Jinmei Ding, Weixing Ye, Jun Wang, Ye Chen, Haiwei Wang, Jing Li, Yi Luo, Jian Su, Yong Huang, Zuohua Liu, Ronghua Dai, Yixiang Shi, He Meng, Qigui Wang

**Affiliations:** 1 Chongqing Academy of Animal Science, Chongqing 402460, P. R. China; 2 Chongqing Engineering Research Center of Goose Genetic Improvement, Chongqing 402460, P. R. China; 3 Department of Animal Science, School of Agriculture and Biology, Shanghai Jiao Tong University; Shanghai Key Laboratory of Veterinary Biotechnology, Shanghai 200240, P. R. China; 4 Shanghai Personal Biotechnology Limited Company, Shanghai 200231, P. R. China

## Abstract

The goose is an economically important waterfowl that exhibits unique characteristics and abilities, such as liver fat deposition and fibre digestion. Here, we report de novo whole-genome assemblies for the goose and swan goose and describe the evolutionary relationships among 7 bird species, including domestic and wild geese, which diverged approximately 3.4~6.3 million years ago (Mya). In contrast to chickens as a proximal species, the expanded and rapidly evolving genes found in the goose genome are mainly involved in metabolism, including energy, amino acid and carbohydrate metabolism. Further integrated analysis of the host genome and gut metagenome indicated that the most widely shared functional enrichment of genes occurs for functions such as glycolysis/gluconeogenesis, starch and sucrose metabolism, propanoate metabolism and the citrate cycle. We speculate that the unique physiological abilities of geese benefit from the adaptive evolution of the host genome and symbiotic interactions with gut microbes.

The goose is a domesticated bird that is reared worldwide and is economically important in central Europe and Asia, especially in China^1^,^2^. Geese supply humans with nutritious meat, large eggs and high-quality liver fat for cooking, as well as soft down and feathers for bedding and clothing^3^. Archaeological evidence indicates that geese might have been domesticated around the Mediterranean Sea ~6,000 years ago^4^ before spreading quickly following patterns of human migration and trade. The evidence also suggests that goose husbandry was common as early as the third millennium BC, in ancient Egypt. During thousands of years of domestication, geese have been considerably shaped by natural and artificial selection. Other than the Yili goose, 25 other breeds of geese found across China, all of which evolved from *Anser cygnoides*^5^.

As important poultry animals, geese exhibit many peculiar characteristics and abilities^6^. For instance, with overfeeding, the goose liver can increase to 5–10 times the weight of a normal liver while the animal remains healthy^7^. Fatty goose liver is a well-known delicacy and a good model for studying human hepatic steatosis, including non-alcoholic fatty liver disease^8^. As a waterfowl species, geese relish grasses but avoid most broad-leaved plants and are therefore suitable for integrated farming systems, as they can be used for weed and pest control for many crops^6^.

It has become a common view in the past few decades that the gut microbiota shows a complementary symbiotic relationship with the vertebrate hosts^9^,^10^. Numerous studies have indicated that the gut microbiome carries out many of the functions of the host, such as metabolism, dietary functions, immune responses, development and physiology^11^,^12^,^13^,^14^, and is associated with the host’s health status and illnesses such as diabetes, obesity, and immune and inflammatory diseases^15^,^16^. Not only is the goose physically suited to the digestion of grass, its gut microorganisms have been proven to be helpful in breaking down grass fibre^17^. However, as there are only a few available studies on herbivorous animals^9^,^10^, the exact mechanisms of interaction between the host and the gut microbiota involved in lipid metabolism and grass fibre digestion remain unclear.

In this study, we report de novo genome assemblies for a domestic goose and a wild goose and comparisons of the gut microbiota between goose and chicken in terms of both the genome and the metagenome. Based on the analysis of sequence data, we addressed the following two aims: (1) obtaining quality genome sequences for both a domestic and a wild goose to illustrate the speciation and adaptive evolution of geese; and (2) integration of information from the genome and metagenome to obtain insight into the mechanism of the interactions between the host and its gut microbes, as related to lipid metabolism and grass fibre digestion, in comparison with the chicken.

## Results

### Summary of the goose genome

To investigate genome structure and evolution, we sequenced and assembled a high-quality genome from a female Sichuan white goose, *Anser cygnoides Linn. var domestica* (‘domestic goose’ hereafter), to 75× coverage, with 91% of the assembly covered at least 20-fold (Table S1). We also re-sequenced one female *Anser cygnoides* (‘wild goose’ hereafter, Fig. S1) to 48× coverage, with 88.4% of the sequence covered more than 20-fold (Table S1). The total of 312,730,302 reads for the domestic goose yielded a draft assembly through integrating the paired-end and mate-pair libraries, while 473,803,082 reads were generated for the wild goose from paired-end libraries (Table S2). The average guanine-cytosine (GC) content of the domestic goose was 41.68% (Table 1), indicating that GC-biased non-random sampling did not strongly affect the assembly. Our assembled genome size was 1,100,859,441 base pairs (bp) (Table 1), which is slightly smaller than the estimated size of 1,198,802,839 bp (Table S3, Fig. S2), with scaffold and contig N50 sizes of 5.1 Mb and 35 kb, respectively (Table 1). The assembled genome size obtained for the domesticated goose is identical to the size reported in a previous study^5^. In comparison with the previous study, our domesticated goose genome exhibits longer contig N50 lengths but shorter scaffold N50 lengths^5^. The average coding sequence (CDS) length was 1,606 bp (Table S4). We detected 6.9% repetitive DNA and 361,510 InDels in the domestic goose genome (Tables S5 and S6) and predicted 12 rRNAs, 204 tRNAs, 223 snoRNAs, 54 snRNAs and 345 other ncRNAs in the genome (Table S7). These results are consistent with previous findings indicating that avian genomes present lower levels of repeat elements than those of other tetrapod vertebrates^18^. This whole-genome shotgun (WGS) project has been deposited at DDBJ/EMBL/GenBank under accession number LABU00000000.

To assist in genome annotation, we performed Illumina RNA sequencing (RNA-seq) of 11 goose tissues: torso, heart, liver, brain, spleen, abdominal fat, pancreas, ovary, duodenum, muscular stomach and lung^19^. We predicted 16,288 protein-coding genes in the domestic goose based on RNA-seq, homology and ab initio gene prediction. Of these genes, 83.13% were functionally annotated according to the BLASTnr, KEGG, KOG and GO databases (Table S8).

### Analysis of genome evolution

**Figure 1:**
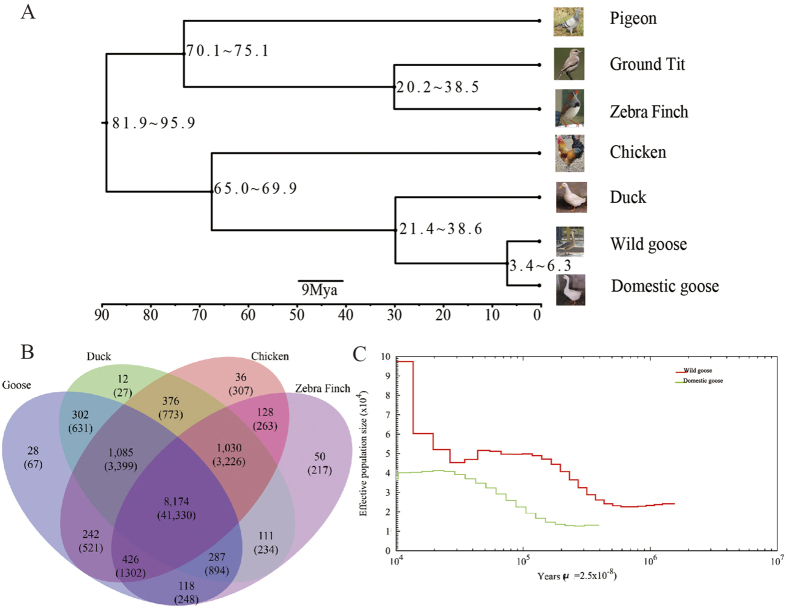
Genomic comparisons between the goose and other bird species. (**A**) Super tree inference for seven birds. The topology was evaluated based on the input tree bootstrap percentages. Distances are shown in millions of years. (**B**) Unique and homologous gene families. The numbers of unique and shared gene families are shown in each of the diagram components, and the total number of gene families for each animal is given in parentheses. (**C**) Demographic history of wild and domestic geese. Reconstructed population demographics of wild and domestic geese for the past 2 million years.

To study goose evolution, we constructed a phylogenetic tree using single-copy genes from the genomes of seven bird species (wild goose, domestic goose, pigeon, ground tit, zebra finch, chicken and duck) (Fig. 1A). According to the phylogenetic tree, wild and domestic geese were clustered into a subclade, and we calculated the divergence time between wild and domestic geese to be approximately 3.4~6.3 Mya, which is consistent with the hypothesis that the domestic goose was domesticated from the wild goose^5^. The divergence time between the goose (wild and domestic) and the duck was estimated to be between 21.4 to 38.6 Mya, and the chicken diverged from the common ancestor of the duck and the goose 65.0~69.9 Mya, which is consistent with previous results^20^. Analysis of the demographic history of population size performed via PSMC revealed the occurrence of a bottleneck event for wild geese approximately 25–45 Kya Following this event, the population size of domestic geese began to steadily increased beginning 350 Kya and has been maintained at approximately 40,000 animals Fig. 1C. We expected that the curves for the two goose species would cross at some point in time because they originated from a common ancestor. However, we failed to trace their demographic histories farther back than 2 Mya. The fact that the curves for wild and domestic geese did not cross over the past 2 million years partially supported the divergence time that we inferred from the phylogenetic analysis.

We constructed families of homologous proteins to detect gene families that have undergone expansion or contraction in goose compared with three other bird species (zebra finch, chicken, and duck). These four species share 8,174 orthologous groups (Fig. 1B). A total of 1,085 clusters containing 3,399 gene models were shared only among the goose, duck, and chicken genomes, while the goose genome exhibited 67 genes in 28 clusters that were not present in the chicken, duck or zebra finch genomes, again demonstrating the evolutionary closeness of these species. In total, we determined that there were 197 expanded and 1,849 contracted gene families in goose compared with the common ancestor of the four species. We identified the rapidly evolving genes in goose versus chicken through nonsynonymous/synonymous (Dn/Ds) analysis. In comparison with chicken, these expanded and rapidly evolving gene families in the goose genome were observed to mainly be involved in metabolism, including energy metabolism, carbohydrate and lipid metabolism, nucleotide and amino acid metabolism, and secondary metabolism. This is consistent with the adaptation of the goose to variable environments, suggesting that the metabolism of geese differs from that of chickens (Table S9).

We also found that genes encoding Na^+^, K^+^-ATPase and epithelial Na^+^, K^+^, H^+^, and 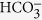 channels had rapidly evolved or expanded in pancreatic beta-cells (insulin secretion, KO 04911), thyroid follicular cells (thyroid hormone, KO 04918), salivary acinar cells (salivary secretion, KO 04970), gastric parietal cells (gastric acid secretion, KO 04971), pancreatic acinar cells and pancreatic duct cells (pancreatic secretion, KO 04972) and cholangiocytes and hepatocytes (bile secretion, KO 04976). The ATP, ATPase, AE2, NBCl and NBC gene families were found to be expanded in the goose genome compared with the chicken. Interestingly, these cells and genes are enriched in the digestive tract, suggesting that geese may be able to reabsorb metabolites more efficiently than chickens (Table S10).

The SNP heterozygote rates of coding and non-coding regions in wild and domestic geese were calculated (Table S11). We found that the overall heterozygosity rate in domestic goose was lower than in wild goose across all genomic regions, which suggests that artificial selection has reduced the genetic diversity of domestic goose^21^.

### Metagenome sequencing of the gut microbiota

We sequenced the V4 regions of 16S rDNA from 56 faecal samples obtained from Sichuan White geese (n = 26) and QingJiaoMa chickens (n = 30). All of the sequences were classified into different operational taxonomic units (OTUs) at 97% similarity.

**Figure 2:**
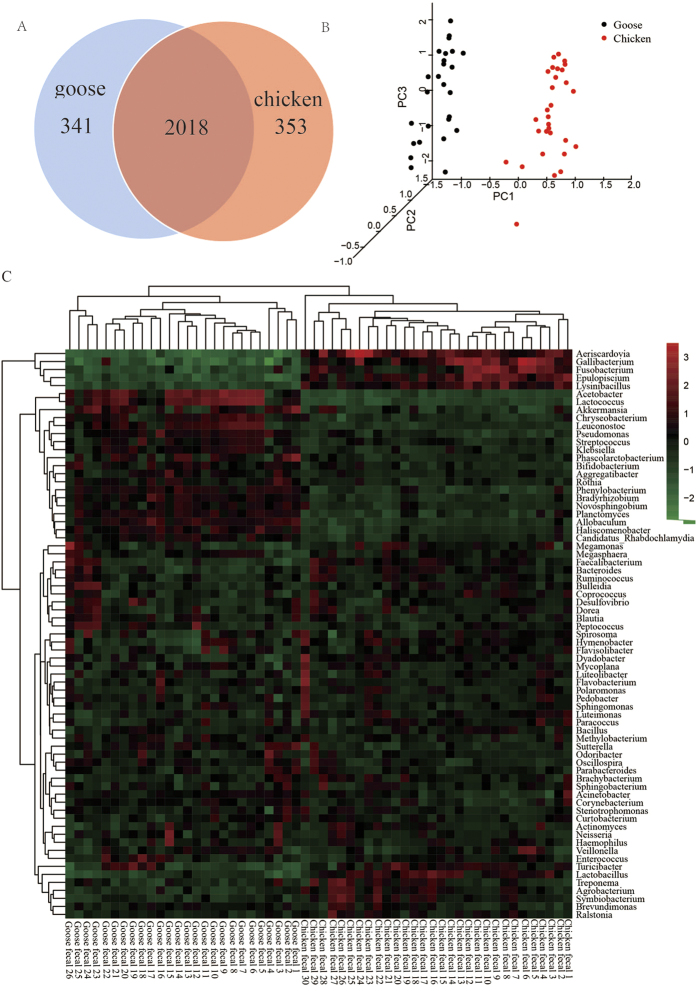
Summary of goose and chicken metagenomes. (**A**) OTUs present in goose and chicken faecal samples. (**B**) Differences in the gut microbiota between goose and chicken according to PCoA. (**C**) Heat map of differences in the gut microbiota between goose and chicken at the genus level.

In total, 1,727,874 sequence reads were obtained from the 56 samples, with an average read length of 224 bp (Fig. S3). The read number per sample ranged from 9,851 to 78,113, averaging 30,664 (Table S12). The rarefaction curves indicated that the sequencing coverage was adequate (Fig. S4). Taxa present in at least two-thirds of the samples were considered common. Among the 2,359 and 2,371 representative OTUs found in goose and chicken, respectively, 2,018 were shared between geese and chickens (Fig. 2A), and 846,491 reads from goose and 881,383 from chicken were used for further analysis (Table S12). To obtain the phylogenetic classifications of the metagenomic reads for each sample, we performed a classification analysis using RDP, aided by the Greengene and SSU databases. The results were assigned to phylum, class, order, family, genus and species levels based on an identity level of 97%. A total of 35 phyla (Table S13), 86 classes (Table S14), 157 orders (Table S15), 281 families (Table S16) and 507 genera (Table S17) were found in the two groups.

To characterize the differences in the compositions of the two groups, we compared the gut microbiota of goose (n = 26) and chicken (n = 30). A clear distinction in the microbiota was revealed through PCoA (Fig. 2B). We employed four indices (the Chao, ACE, Simpson and Shannon indexes) to estimate the alpha diversity of the goose and chicken faecal samples. The Chao and ACE indexes were lower in goose than in chicken faecal samples, and there were highly significant differences (P < 0.01) between the groups, according to *T* test statistics (Table S18). However, the Simpson and Shannon indices were higher for faecal samples from goose than those from chicken, but the difference between the two groups was not significant (Table S18). These results suggested that the richness of the gut microbe in goose faecal material was significantly lower than that in chickens, and the diversity of the gut microbiota of geese was slightly higher than in chickens.

At the phylum level, the predominant bacterial phyla in all of the samples from the two groups were *Firmicutes* and *Proteobacteria*. Compared with Sichuan White goose (34.8% for Firmicutes, 34.7% for *Proteobacteria*), Qingjiaoma chicken exhibited a higher proportion of Firmicutes (61.1%) but a lower proportion of *Proteobacteria* (21.8%) (Fig. S5A,B). At the genus level, *Haliscomenobacter, Lactobacillus* and *Streptococcu*s were the dominant groups in goose, while *Blautia, Lactobacillus,* and *Haliscomenobacter* were the dominant groups in chicken (Fig. S5C,D). Most of the dominant microbiota found in geese were different from those of chickens. In summary, the composition and abundance of the microbiome community were different between goose and chicken, except in the genetic base, suggesting that the composition of the microbiome community is mainly associated with the food intake strategy (diet: goose 220 g, chicken 100 g; grass: goose: 120 g, chicken: 20 g; for 20 days).

To determine the differences in the composition and relative abundance of the microflora in the microbiomes of these two domesticated avian species at the genus level, we considered a difference in relative abundance to exist if (i) there was a two-fold difference between the mean relative abundance of each genus in each sampled population; or (ii) the difference in the mean relative abundance was significant based on a false discovery rate corrected P-value threshold of <0.05; or (iii) the average number was above thirty. A total of 52 significantly different genera were identified between the goose and chicken groups (Fig. 2C, Table 2). Among these genera, *Lactobacillus, Streptococcus, Lactococcus, Clostridium, Peptococcus, Bifidobacterium* and *Ruminococcus* were significantly different between goose and chicken (Fig. 2C). These groups of bacteria ferment carbohydrates and proteins and produce short-chain fatty acids (SCFAs) (butyrate, acetate, lactate, propionate, valerate, and isovalerate)^22^. The microflora of the goose was similar to that of the human large intestine or the rumen fermentation mixture formed by individual groups of anaerobic bacteria^23^, suggesting that the SCFAs differed between goose and chicken. These results are consistent with previous findings^24^,^25^.

### Integrated analysis of the host genome and the gut metagenome

In this study, we analysed the expanded and rapidly evolving gene families in the goose genome and the differences in bacterial composition between goose and chicken faecal samples to identify potential evolutionary events that might be related to adaptive evolution. The results showed that the expanded and rapidly evolving gene families in the goose genome are mainly associated with metabolic functions (Fig. S6), including nucleotide metabolism, amino acid metabolism, lipid metabolism and carbohydrate metabolism and energy metabolism (Fig. S7). Different bacterial groups were also mainly involved in metabolism (44.98%) (Fig. S8), including energy metabolism, amino acid metabolism, carbohydrate metabolism and metabolism of other amino acids (Fig. S9). We found both expanded and rapidly evolving genes and different bacterial groups to be enriched in amino acid metabolism and carbohydrate metabolism pathways (Tables S19 and 20). However, we established that few expanded and rapidly evolving genes, but many differential bacterial groups displayed significantly enrichment for the biodegradation and metabolism of xenobiotics (Table S21).

**Figure 3:**
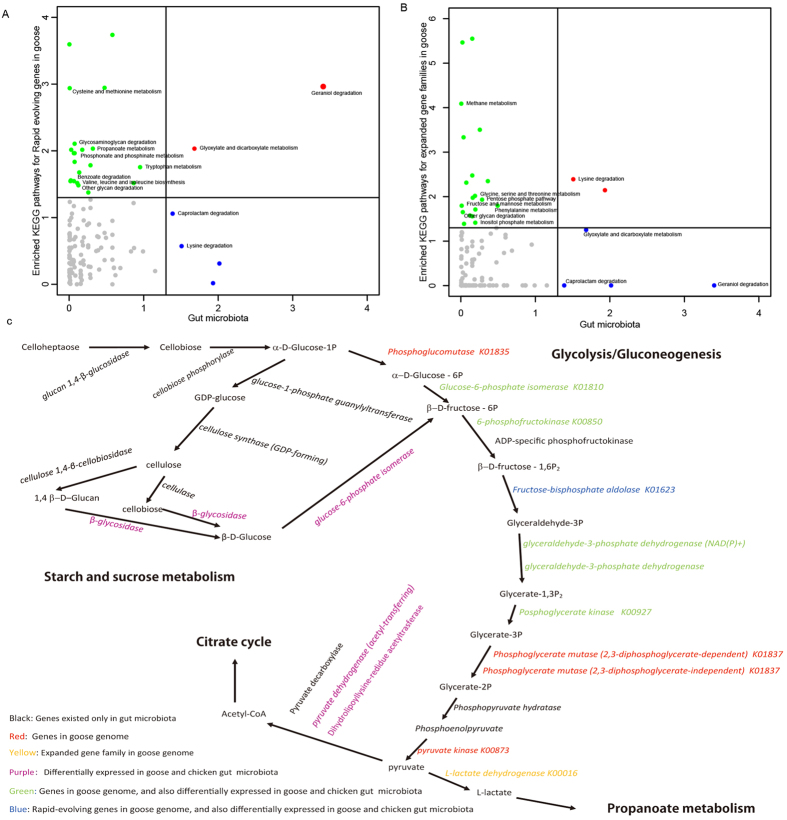
Comparison of the gene pathways of the host genome and the gut microbiota. (**A**) Enriched KEGG pathways of rapidly evolving genes and gut microbiota that are differentially represented in goose and chicken. (**B**) Enriched KEGG pathways of expanded gene families and gut microbiota that are differentially represented in goose and chicken. (**C**) Integrated analysis of gene pathways between the host genome and gut microbiota.

In geese, the high capability to digest fibre-rich feed is quite notable. As shown in Fig. 3A,B, ‘other glycan degradation’ was a significantly enriched KEGG pathway among both the rapidly evolving genes and the expanded gene families. As the main component of grass fibres (cellulose) is a glycan, and considering the existence of several other carbohydrate metabolism pathways (such as ‘pentose phosphate’ and ‘fructose and mannose metabolism), these results suggest that the goose genome potentially enables better digestion and absorption of this polysaccharide-based feed source. However, the composition of the gut microbiota indicates a clear pathway from cellulose to pyruvate before entering the tricarboxylic acid (TCA) cycle, as shown in Fig. 3C.

Most animals lack the ability to degrade and digest cellulose, and the goose is no exception; however, certain species are capable of digesting cellulose because of their gut microbiota, such as the termite^9^,^10^. Based on our data, we speculate that cellulose is first degraded into cellobiose by cellulase, which exists only in intestinal bacteria. Cellobiose can then enter the glycolysis/gluconeogenesis pathway through two alternative routes. One of these pathways first involves digestion into β-D-glucose by β-glucosidase^26^,^27^, followed by transformation into β-D-fructose-6P by glucose-6-phosphate isomerase^28^,^29^. Notably, the expression of both the β-glucosidase and glucose-6-phosphate isomerase genes has been found to be significantly higher in the intestinal bacteria of geese compared with those of chickens^30^,^31^,^32^,^33^. The other route from cellobiose to β-D-fructose-6P is first involves transformation into α-D-glucose-1P by cellobiose phosphorylase^34^,^35^, followed by transformation into α-D-glucose-6P by phosphoglucomutase^36^ and, finally, transformation into β-D-fructose-6P by glucose-6-phosphate isomerase^29^. Cellobiose phosphorylase was only found in the gut microbiota, while phosphoglucomutase was identified in the goose genome, and glucose-6-phosphate isomerase was also present in the goose genome and was expressed in the intestinal bacteria of geese significantly more highly than in those of chickens. After entering the glycolysis/gluconeogenesis pathway, β-D-fructose-6P can eventually be transformed into pyruvate, catalysed by a series of enzymes encoded by genes either in the host genome or that are expressed by the gut microbiota acting in concert (Fig. 3C). Several of these enzymes, including 6-phosphofructokinase, glyceraldehyde-3-phosphate dehydrogenase and phosphoglycerate kinase, are not only found in the goose genome, but are also expressed at significantly higher levels in the intestinal bacteria of geese than in those of chickens. Fructose-bisphosphate aldolase is an expanded gene family in the goose genome, for which we identified 3 copies in our analysis. Phosphopyruvate hydratase is not found in the goose genome but is expressed by the gut microbiota. Furthermore, two genes that can convert pyruvate into acetyl-CoA (pyruvate dehydrogenase and dihydrolipoyllysine-residue acetyltransferase) were also significantly more highly expressed in goose than in chicken gut microbiota.

## Discussion

In this study, we generated high-quality genome sequences through de novo assembly and deep resequencing, and elucidated the adaptive evolution and divergence time of a domestic and wild goose genome. Our method offers greater robustness than previous studies that have analysed differences in the origins and genetic differentiation of these taxa based on mitochondrial DNA polymorphisms of geese^5^,^37^.

Chickens are closely related poultry species to geese. However, geese are herbivorous waterfowl, and their diet is different from that of chickens, as geese exhibit specialized digestion physiology and can digest dietary fibre. The effects of dietary fibre on the physiological functions of the digestive tract can vary widely, including influencing digestive tract movement, passage time, growth, and enzyme secretion and the physical and chemical characteristics and mechanisms of action of microorganism groups in the digestive tract. We found that many expanded and rapidly evolving gene families displayed metabolic functions and were enriched in the goose genome, but were not significantly different between the gut microbiota of chicken and goose (Fig. 3A,B). Integrated analysis of the host genome and the gut metagenome provided new insight into the molecular characteristics of the herbivorous and lipid metabolism, revealing a network of genes involved in Glycolysis/glycogenesis, beta oxidation, glucose uptake, lipid metabolism and SCFA production, which suggests that geese and their gut microbiota complement each other allowing the digestion of grass fibre, and that symbiotic interactions exist between the host and its gut microbes. Further work will be needed to clarify these connections and explore possible links related to concomitant evolutionary changes in the functional genes of geese and the goose gut microbiota.

## Methods

### Sampling, genome sequencing and assembly

For the domestic goose, 2 ml blood was collected from the wing vein of a 2-year-old female Sichuan White goose named “Wang” provided by the Poultry Science Institute, Chongqing Academy of Animal Science, P. R. China. For the wild goose, the blood sample was collected from a 3-year-old wild goose (*Anser cygnoides*) provided by the Silamulun Zoo of Tong Liao, Inner Mongolia, P. R. China (Fig. S1).

Genomic DNA was extracted from the blood samples using the AxyPrep Blood Genomic DNA Miniprep Kit (Axygen Biosciences, Union City, CA 94587, USA) according to the manufacturer’s protocol. The concentration and molecular size of the DNA were measured using a TBS-380 Mini-Fluorometer (Turner BioSystems, California, USA) and through 1.0% agarose gel electrophoresis.

The protocol employed in this study was reviewed and approved by the Research Ethics Committee and the Animal Ethical Committee of the Chongqing Academy of Animal Sciences. All methods used in this study were performed in accordance with protocols approved by the Laboratory Animal Management Committee of the Chongqing Academy of Animal Sciences and the Ministry of Science and Technology of the People’s Republic of China (Approval number: 2006-398).

For the domestic goose genome, de novo sequencing was performed on the Illumina MiSeq and HiSeq 2000 platform with paired-end libraries and mate-paired libraries. Four paired-end libraries with targeted insert sizes of 400 bp, 400 bp, 700 bp and 700 bp were constructed using the TruSeq Nano DNA LT Library Prep Kit (Illumina, USA), and three mate-paired libraries (2 kb, 5 kb and 10 kb) were constructed using the Nextera Mate Pair Sample Prep Kit (Illumina, FC-132-1001, USA) according to the corresponding manuals. The wild goose genome was resequenced on the Illumina HiSeq 2000 platform with an insert size of 400 bp using a paired-end library.

After removing repeat sequences, adapter sequences, and sequences shorter than 50 bp or those that contained more than three uncertain bases in the raw data, we assembled the domestic goose genome from the high-quality reads using De Novo Assembler Software (Newbler, version 2.8). The size of the goose genome was evaluated using the paired-end libraries via K-mer analysis (K = 17)^38^. Information on the sequence overlap of the paired-end libraries was employed to construct contigs, which were assembled into a scaffold using the information from the paired-end and mate-pair libraries. Finally, intra-scaffold gaps were closed using “Gapcloser” (http://soap.genomics.org.cn/soapdenovo.html). After assembly, we evaluated the completeness of the goose genome assembly using Core Eukaryotic Genes Mapping Approach software (CEGMA), which compared a set of 248 core eukaryotic genes to the assembled sequence. We estimated sequencing coverage and GC content using SOAPaligner by aligning all of the raw reads to the sequence of the scaffold. The average coverage depth was estimated by calculating the depth of each base. The scaffolds were subjected to searches against the NCBI nucleotide databases of fungi and bacteria to check for contaminated sequences, applying the criteria of a BLASTn hit e-value below 1e-5 and an alignment length greater than 50% of the entire length.

### Genome annotations

Protein-coding genes were predicted using three strategies: ab initio prediction, homology-based annotation and a transcriptome-based method. Ab initio prediction was performed using Augustus software (version 2006-08-28) with the parameters trained using predicted homologous proteins^39^. Based on these training genes, SNAP (version 3.0.1) and GLIMMERHMM (version 2006-08-28) estimated the parameters and predicted gene models^40^. To reduce false positives, only de novo predictions that were supported by both methods were taken into consideration for subsequent analyses. The protein repertoires from several sequenced avian species, including *Anas platyrhynchos*, *Ficedula albicollis*, *Meleagris gallopavo*, *Taeniopygia guttata* and *Gallus gallus*, were aligned to the goose genome using Exonerate software (version 2.2.0). The most similar homologous regions were selected using Genewise to define the gene models. Moreover, we aligned the transcriptome reads from 11 goose tissues^19^,^41^ to the goose genome using PASA (version r20140417) to identify exon regions and splicing sites^42^, providing further evidence for the homology-based prediction. Finally, we merged the results of the three methods using EvidenceModeler.

Gene functions were assigned according to the best match of the alignment against the SwissProt database using BLASTALL software with a cut-off e-value of 1e-6. Motifs and domains were annotated through searches against publicly available databases, including Pfam, PRINTS, PROSITE, ProDom, and SMART, using InterProScan. Gene Ontology (GO) terms were obtained from the Interpro database by BLAST2GO software. KEGG annotation was performed by the KAAS online server using the SBH method against the species set, while KOG annotation was determined by BLASTp against the KOG database with a cut-off e-value of 1e-5. Known transposable elements (TEs) were identified by searching against the nucleotide repetitive database and the protein repetitive database of Repbase (version 20140131)^43^ using RepeatMasker software (version 4.0.5)^44^. Furthermore, a de novo goose repeat library was constructed using RepeatModeler software. Tandem repeats were annotated with RepeatScout using default parameters, including satellites, low complexity repeats, simple repeat and high and medium copy repeats (>10 copies).

tRNAs and rRNA genes were identified using tRNAscan-SE (Version 1.3.1) with eukaryotic parameters and RNAmmer (Version 1.2)^45^, respectively. microRNAs (miRNAs) and small nuclear RNAs (snRNAs) were identified by searching against the Rfam database^46^.

### Evolutionary and comparative genome analysis

To gain insight into the evolution of goose gene families, we reconstructed single-copy genes via the orthomcl method from the sequenced genomes of the following 7 bird species: wild goose, domestic goose, pigeon, ground tit, zebra finch, chicken, and duck^47^. We then subjected the single-copy genes to BLAST searches against all genomes using Muscle software (version 3.8.425), applying the default search parameters^48^. We selected the optimum amino acid model to construct gene family trees using the PHYML software (version 3.2)^49^. The divergence times of the species were estimated with the MCMCTree program of PAML (version 4.7) software^50^. The demographic histories of domestic and wild geese were inferred via “pairwise sequentially Markovian coalescence” (PSMC). The parameter settings were as follows: −N30 −t15 −r5 −p 4 + 25*2 + 4 + 6. The generation times of domestic and wild geese were set to 1 and 3 years, respectively. The neutral mutation rate per generation (μ) was set to 2.5* 10^−8^.

The goose gene families were constructed using TreeFam to investigate the orthology relationships between goose and three other species (*Gallus gallus, Anas platyrhynchos,* and *Taephila guttata*). CAFE (version 3.1) was employed to detect gene families that have undergone expansion or contraction in the goose compared with other species. This software uses a stochastic model of gene birth and death to infer statistically significant gains and losses in gene families, employing a phylogenetic tree and a table of gene copy numbers in each organism. A family-wide significance threshold of 0.05 was applied. We checked the candidate families detected by CAFE to filter out artefacts.

The BWA program (Fast and accurate short read alignment with Burrows) was employed to remap the useful reads from wild goose to the assembled scaffold for domestic goose with default parameters. Reads that could map to multiple positions were removed in the subsequent analysis. The SAMtools pipeline (sequence alignment/map (SAM) format and SAMtools) was used to retrieve SNPs and small indels (<50 bp) with default settings. We flagged a candidate SNP as a likely false positive if it fulfilled the following criteria: (1) total depth above 400 or below 10; (2) root mean square of mapping quality below 20; (3) depth of alternate bases below 4; (4) P-value of reference and non-reference bases evenly distributed on both strands below 1*10^−4^ (Fisher exact test). These thresholds were applied to both the heterozygous SNPs within the wild and domestic goose genomes and the homozygous SNPs between them. The heterozygosity rate was estimated using sliding windows of 10 kb with 90% overlap between adjacent windows. The w2-test was performed for each window to identify the regions where the heterozygosity rate of the domestic goose was significantly lower than that of the wild goose (P < 0.05 after Bonferroni correction).

### Gut microbial 16S rDNA sequencing

At an age of 160 days, fresh faecal samples were randomly obtained from 26 Sichuan White geese (14 males, 12 females) and 30 QingJiaoMa chickens (15 males, 15 females). These 56 individuals were randomly sampled from the larger population of the same generation, which had been given the same diet (Table S22) and maintained under the same husbandry conditions. Microbial genomic DNA was extracted from faecal samples using the QIAamp DNA stool mini kit (QIAGEN, cat#51504) following the manufacturer’s recommendations. The V4 hypervariable regions of 16S rRNA were amplified through PCR using the barcoded fusion primers we described in a previous report^51^. The 16S rDNA of faecal microbes was sequenced using the Illumina MiSeq platform and trimmed using a 5 bp sliding window with 1 bp-length steps based on the phred algorithm^52^,^53^. We discarded sequence reads of less than Q20 and those with a length of less than 150 bp as well as those that contained ambiguous bases or showed an average phred score lower than 25, a homopolymer run exceeding 6, mismatches in primers, or a length shorter than 100 bp. Sequences that overlapped the region between R1 and R2 without any mismatches for at least 10 bp were assembled according to their overlapping sequences. After trimming, we merged the sequence reads using Flash (v1.2.6) (http://www.genomics.jhu.edu) with the criteria that the overlap of the assembled reads must be more than 10 bp without mis-assembly. Merged fastq files were converted to fasta files and exported into Quantitative Insights into Microbial Ecology (QIIME) software^54^ to identify the sequence reads of individual samples. To improve the accuracy of the results, we identified and removed chimeric sequences using UCHIME^55^ in mothur (version 1.31.2, http://www.mothur.org/)^56^ and discarded sequences that exhibited the following characteristics: read length <200 bp, ambiguous base calling, six-base homopolymer runs, lack of primers, primer mismatches, or uncorrectable barcodes. After sample assignment, the forward primer and barcode sequences of the reads were removed.

### Taxonomic classification and comparative analysis

The remaining sequences were clustered into OTUs using the seed-based uclust algorithm with a cutoff of 97%^57^. Taxonomic identification was assigned using the RDP classifier^58^ in QIIME with a confidence threshold of 0.8. The longest sequences from each OTU were subjected to BLAST searches against the Greengene bacterial 16S rRNA database at a minimum e-value threshold of 0.001 using the best hit classification option to classify the abundance count of each taxon^59^. The taxa showing differences in abundance between groups were evaluated at the genus and phylum levels using Metastats^60^, with P values corrected via multiple hypothesis testing using the false discovery rate (FDR). The resultant OTU files were imported into the MEtaGenome Analyzer (MEGAN)^61^ program for taxonomic analysis and assignment of the amplicon sequence data. The size and colour of each node label is proportional to the number of sequence reads for groups at each taxonomic level. To investigate the differences between the microbial communities of goose and chicken, we performed weighted (based on the abundance of taxa) and unweighted UniFrac (sensitive to rare taxa)^62^ (http://bmf2.colorado.edu/unifrac/) tests to measure the pair-wise phylogenetic distances of the three groups. A principal coordinate analysis (PCoA) was computed from the resulting distance matrices to compress dimensionality into 3D PCoA plots^63^, enabling visualization of the relationships of the samples. We generated the rarefaction curve for each individual sample to estimate species richness (Chao1, ACE), alpha diversity (Simpson, Shannon), and whole-tree phylogenetic diversity with respect to sequence depth using QIIME and mothur^54^.

### Prediction of microbial functions

We predicted the functional profiles of the bacterial metagenomes in the two groups based on the relative abundance of individual OTUs using PICRUSt (http://picrust.github.io/picrust/)^64^. The OTUs were mapped to the gg13.5 database at 97% similarity using the QIIME command “pick_closed_otus”. The OTU abundance was automatically normalized using 16S rRNA gene copy numbers from known bacterial genomes in Integrated Microbial Genomes (IMG). The predicted Kyoto Encyclopedia of Genes and Genomes (KEGG) orthologues were summarized to level-3 functional categories and compared among groups using the Statistical Analysis of Metagenomic Profile package STAMP (http://kiwi.cs.dal.ca/Software/STAMP)^65^. Differentially represented gene families were identified using the two-sided Welch’s t-test with Storey’s false discovery rate correction.

## Additional Information

**How to cite this article**: Gao, G. *et al*. Genome and metagenome analyses reveal adaptive evolution of the host and interaction with the gut microbiota in the goose. *Sci. Rep.* **6**, 32961; doi: 10.1038/srep32961 (2016).

## Supplementary Material

Supplementary Information

## Figures and Tables

**Table 1:** Characteristics of the domestic goose genome assembly.

Property	Contigs	Scaffold
Sequences greater than 1 kb	64,978	1,837
Shortest (bp)	363	2,004
Longest (bp)	399,111	20,207,557
N20	72,765	8,949,512
N50	35,032	5,103,766
N90	8,519	1,057,331
Total sequence length	1,100,859,441	1,100,859,441
GC content	41.68%	41.68%

**Table 2:** Differential gut microbiota in goose and chicken.

Genus	Goose	Standard error	Chicken	Standard error	p-value	q-value
*Akkermansia*	5.2230%	0.022253	0.3429%	0.001666	0.001998	0.003863
*Enterococcus*	4.3680%	0.014875	0.7152%	0.000539	0.000999	0.002138
*Acetobacter*	3.9694%	0.009377	0.0074%	0.000024	0.000999	0.002138
*Lactococcus*	2.9913%	0.005343	0.0046%	0.000017	0.000999	0.002138
*Streptococcus*	2.8983%	0.004834	0.4156%	0.000427	0.000999	0.002138
*Klebsiella*	1.9739%	0.002125	0.2828%	0.000543	0.000999	0.002138
*Planctomyces*	1.3184%	0.00322	0.0982%	0.00025	0.000999	0.002138
*Phenylobacterium*	1.1830%	0.002202	0.0950%	0.000279	0.000999	0.002138
*Pseudomonas*	0.9606%	0.003407	0.0868%	0.000328	0.000999	0.002138
*Sutterella*	0.6698%	0.002271	0.0896%	0.000534	0.003996	0.006822
*Phascolarctobacterium*	0.4943%	0.002739	0.0123%	0.000061	0.001998	0.003863
*Allobaculum*	0.4785%	0.001035	0.0054%	0.000022	0.000999	0.002138
*Ruminococcus*	0.4334%	0.001551	0.0946%	0.000393	0.046953	0.057961
*Peptococcus*	0.3625%	0.001795	0.0195%	0.000064	0.003996	0.006822
*Chryseobacterium*	0.3571%	0.000472	0.0385%	0.000156	0.000999	0.002138
*Bifidobacterium*	0.2951%	0.001144	0.0313%	0.000123	0.000999	0.002138
*Leuconostoc*	0.2621%	0.000459	0.0016%	0.00001	0.000999	0.002138
*Haliscomenobacter*	0.2446%	0.000541	0.0051%	0.000025	0.000999	0.002138
*Bradyrhizobium*	0.2362%	0.000494	0.0123%	0.000041	0.000999	0.002138
*Rothia*	0.1850%	0.000829	0.0103%	0.00003	0.000999	0.002138
*Blautia*	0.1540%	0.000392	0.0249%	0.000097	0.001998	0.003863
*Novosphingobium*	0.1508%	0.000348	0.0247%	0.000147	0.000999	0.002138
*Gemmata*	0.1331%	0.000388	0.0056%	0.000021	0.000999	0.002138
*Aggregatibacter*	0.1232%	0.000497	0.0101%	0.000043	0.000999	0.002138
*Nelumbo*	0.1155%	0.000284	0.0129%	0.000049	0.000999	0.002138
*Brevibacterium*	0.1004%	0.000388	0.0212%	0.000051	0.013986	0.020591
*Sediminibacterium*	0.0908%	0.00016	0.0025%	0.00001	0.000999	0.002138
*Wautersiella*	0.0784%	0.000137	0.0162%	0.000135	0.000999	0.002138
*Rhodoplanes*	0.0745%	0.000185	0.0055%	0.00002	0.000999	0.002138
*Rhodobacter*	0.0740%	0.000185	0.0096%	0.000026	0.000999	0.002138
*Micrococcus*	0.0721%	0.00022	0.0083%	0.000036	0.000999	0.002138
*Staphylococcus*	0.0711%	0.000197	0.0145%	0.000036	0.002997	0.005465
*Methyloversatilis*	0.0676%	0.000143	0.0111%	0.000034	0.000999	0.002138
*Propionibacterium*	0.0543%	0.000366	0.0042%	0.000023	0.005994	0.009716
*Bdellovibrio*	0.0350%	0.000104	0.0007%	0.000004	0.000999	0.002138
*Burkholderia*	0.0319%	0.000076	0.0040%	0.000015	0.000999	0.002138
*Dehalobacterium*	0.0252%	0.000085	0.0006%	0.000003	0.000999	0.002138
*Dok59*	0.0147%	0.000029	0.0008%	0.000006	0.000999	0.002138
*Nitrospira*	0.0064%	0.000019	0.0004%	0.000003	0.000999	0.002138
