# Supplementary material for: Genome and metagenome analyses reveal adaptive evolution of the host and interaction with the gut microbiota in the goose
Source: Sci Rep. 2016 Sep 9;6:32961. doi: 10.1038/srep32961 (PMC5016989; doi:10.1038/srep32961)
Supplement: Supplementary Information [file srep32961-s1.doc]

Supplementary Figures and Tables for

Genome and metagenome analyses reveal adaptive evolution of the host and interaction with the gut microbiota in the goose

Guangliang Gao^1,2^, Xianzhi Zhao^1,2^, Qin Li^1,2^, Chuan He^3,4^, Wenjing Zhao^3^, Shuyun Liu^3^, Jinmei Ding^3^, Weixing Ye^4^, Jun Wang^4^, Ye Chen^4^, Haiwei Wang^1,2^, Jing Li^1,2^, Yi Luo^1,2^, Jian Su^1^, Yong Huang^1^, Zuohua Liu^1^, Ronghua Dai^3^, Yixiang Shi^4^, He Meng^3^,*, Qigui Wang^1,2^,*

^1^Chongqing Academy of Animal Science, Chongqing 402460, P. R. China.

^2^Chongqing Engineering Research Center of Goose Genetic Improvement, Chongqing 402460, P. R. China.

^3^Department of Animal Science, School of Agriculture and Biology, Shanghai Jiao Tong University; Shanghai Key Laboratory of Veterinary Biotechnology, Shanghai 200240, P. R. China.

^4^Shanghai Personal Biotechnology Limited Company, Shanghai 200231, P. R. China

*Corresponding Author: He Meng (menghe@sjtu.edu.cn); Qigui Wang (wangqigui@hotmail.com)

Supplementary Figures


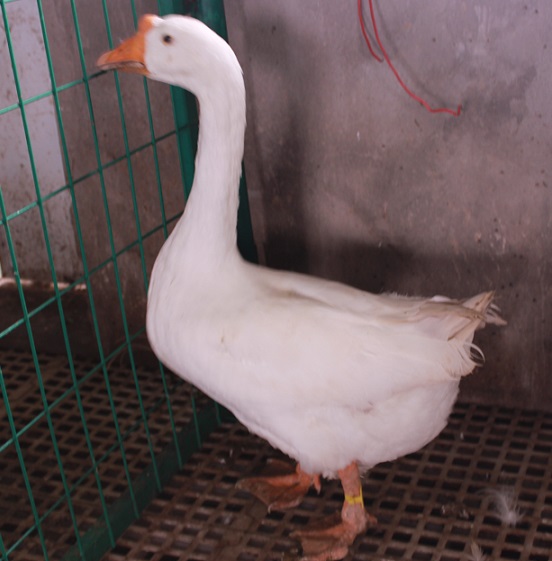

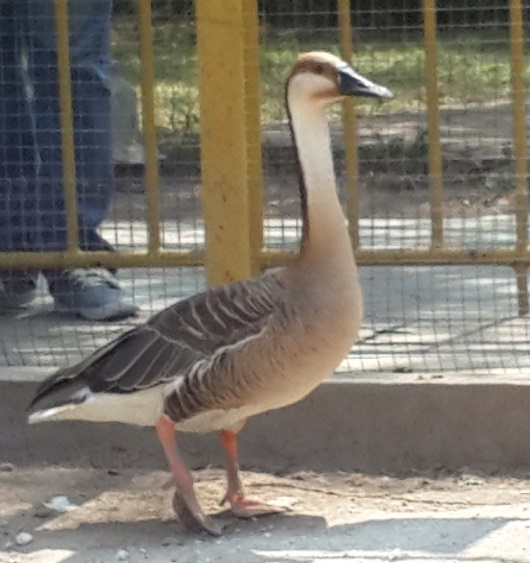


1. Sichuan White goose B. Anser cygnoides

Figure S1. The domestic goose and wild goose sequenced in this study.

1. a 2-year-old female Sichuan White goose named “Wang” provided by poultry science institute, Chongqing Academy of Animal Science, P. R. China.
2. a 3-year-old wild goose (Anser cygnoides) provided by Silamulun Zoo of Tong Liao, Inner Mongolia, P. R. China.


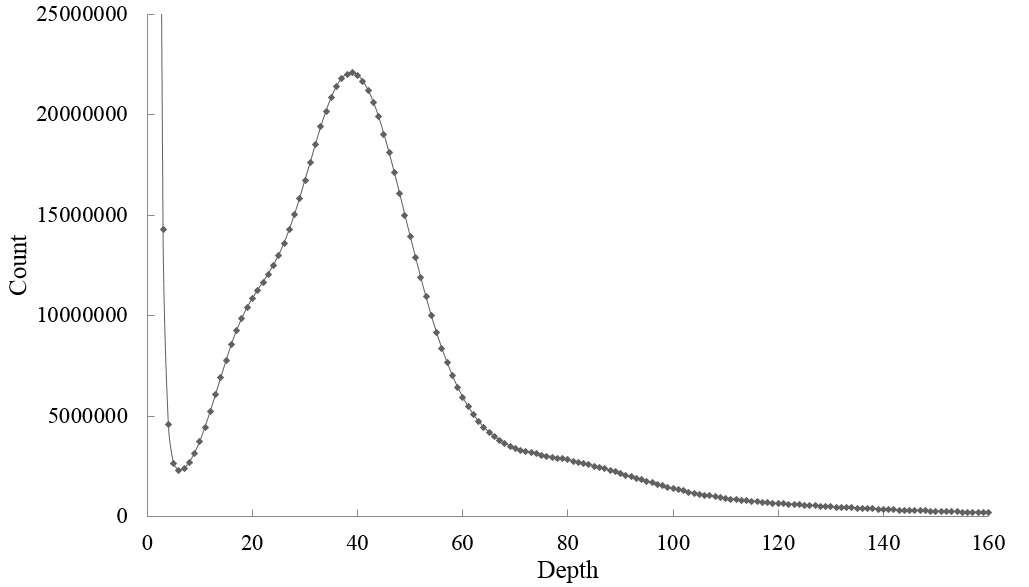
 Figure S2. Distribution of 17-mer frequency in the corrected PE reads.


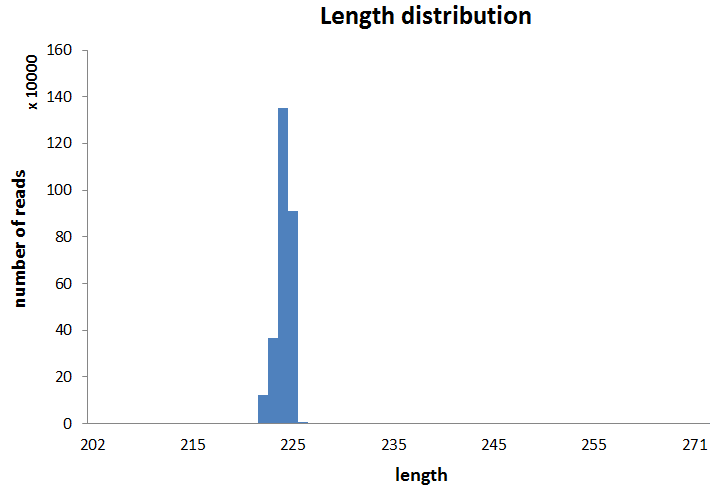
Fig S3 length distribution of all metagenomic reads


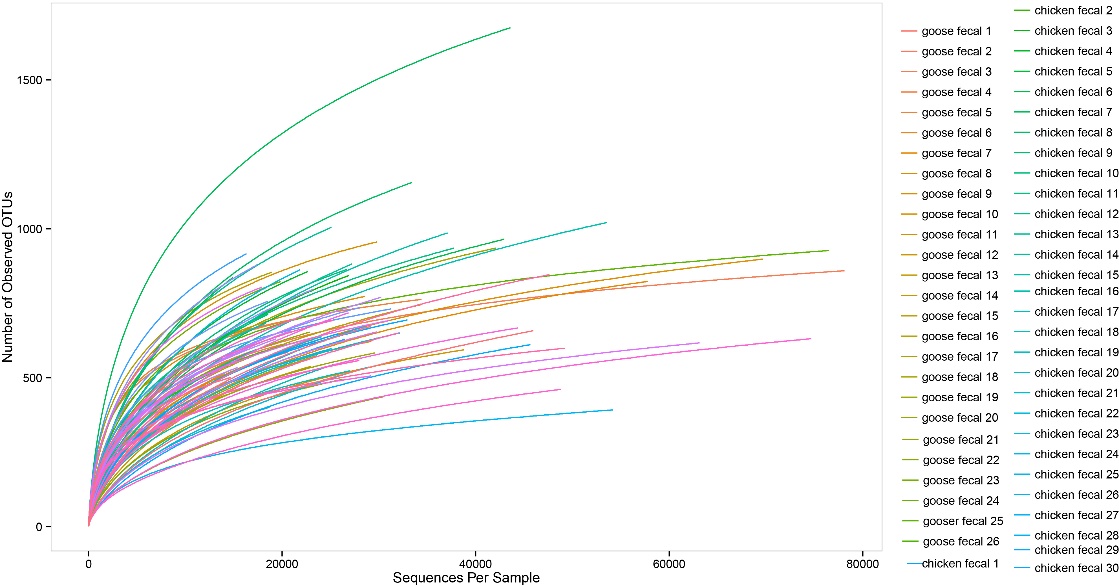


Fig S4 Rarefaction Curve of all samples under the condition of 0.97 similarity

­­­­
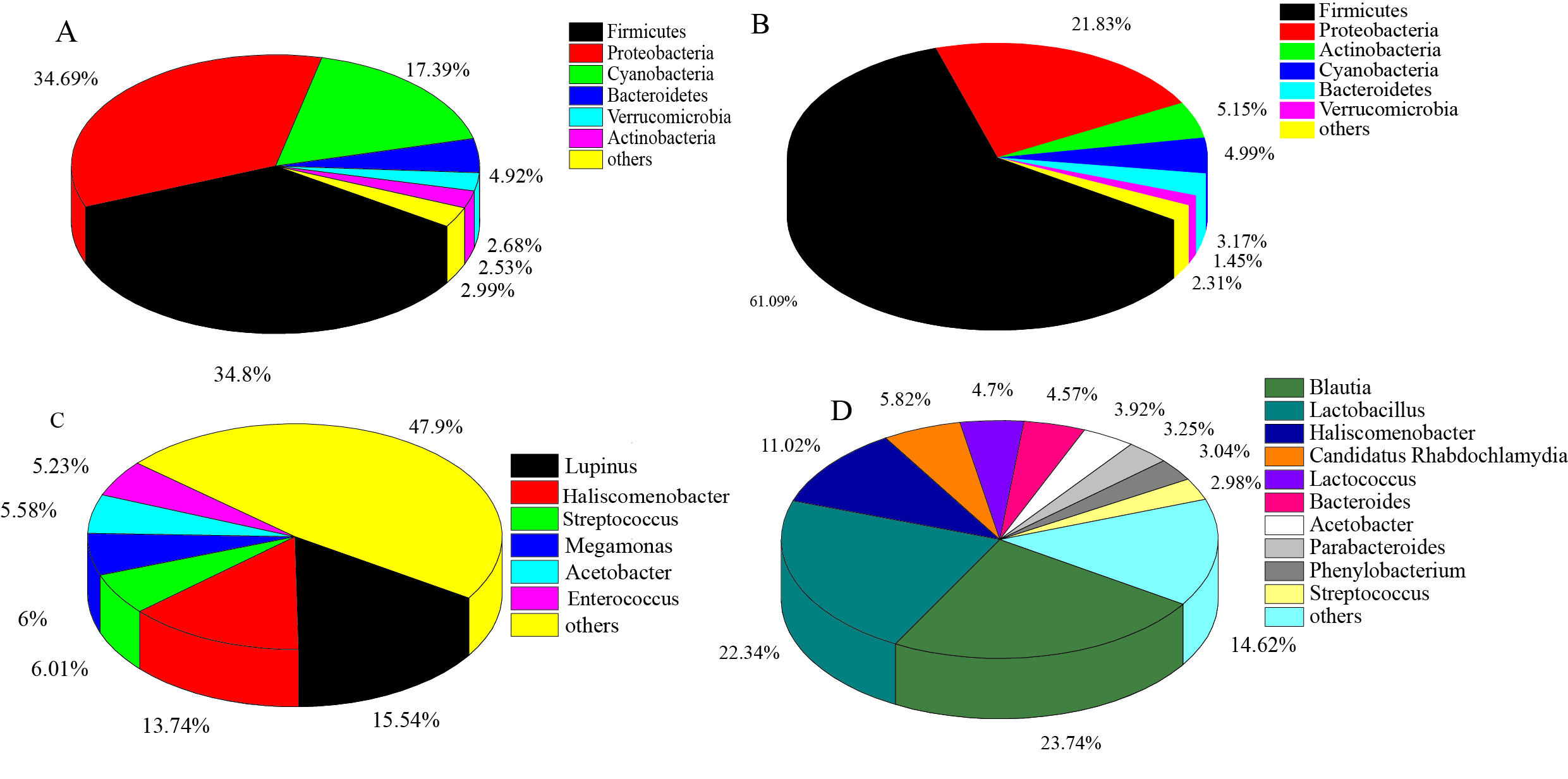


Fig S5 Profiles of gut microbes in goose and chicken feces at the rank of phylum and genus. a, Composition structure of microbiome in feces at goose feces of phylum. b, Composition structure of microbiome in feces at chicken feces of phylum. c, Composition structure of microbiome in feces at goose feces of genus. d, Composition structure of microbiome in feces at chicken feces of genus.


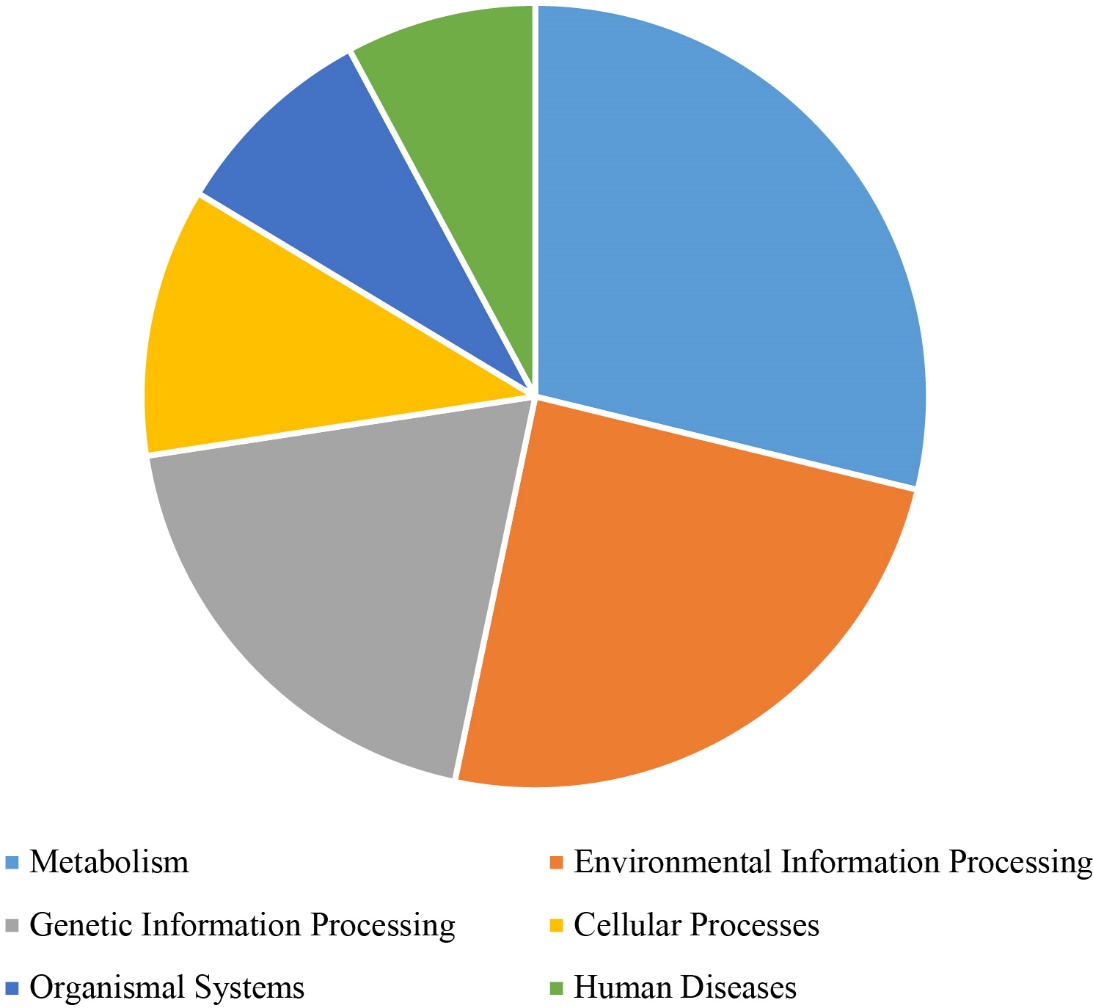


Fig S6 Profiles of expanded and rapid evolving gene families of goose genome in KEGG class A


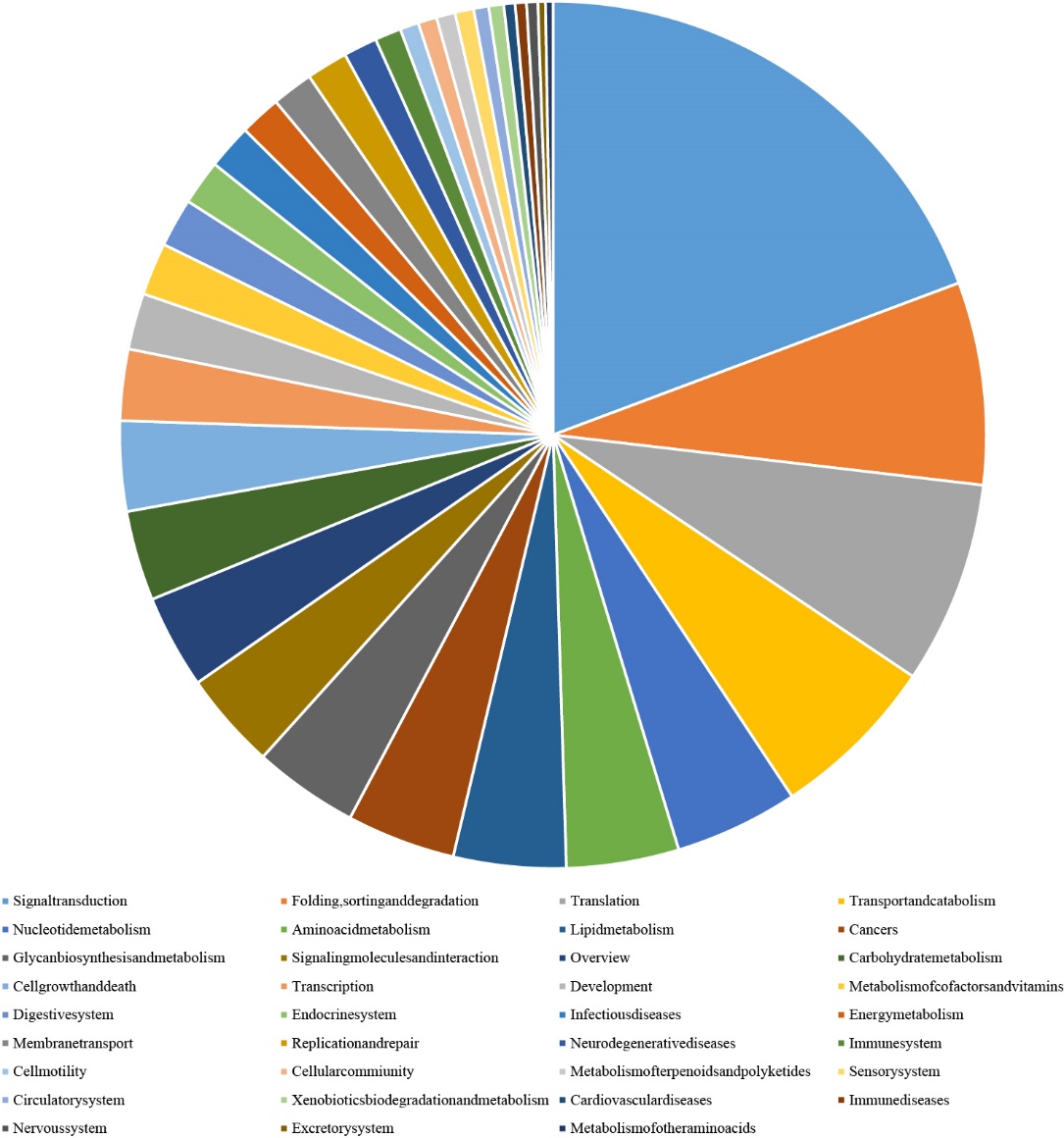


Fig S7 Profiles of expanded and rapid evolving gene families of goose genome in KEGG class B


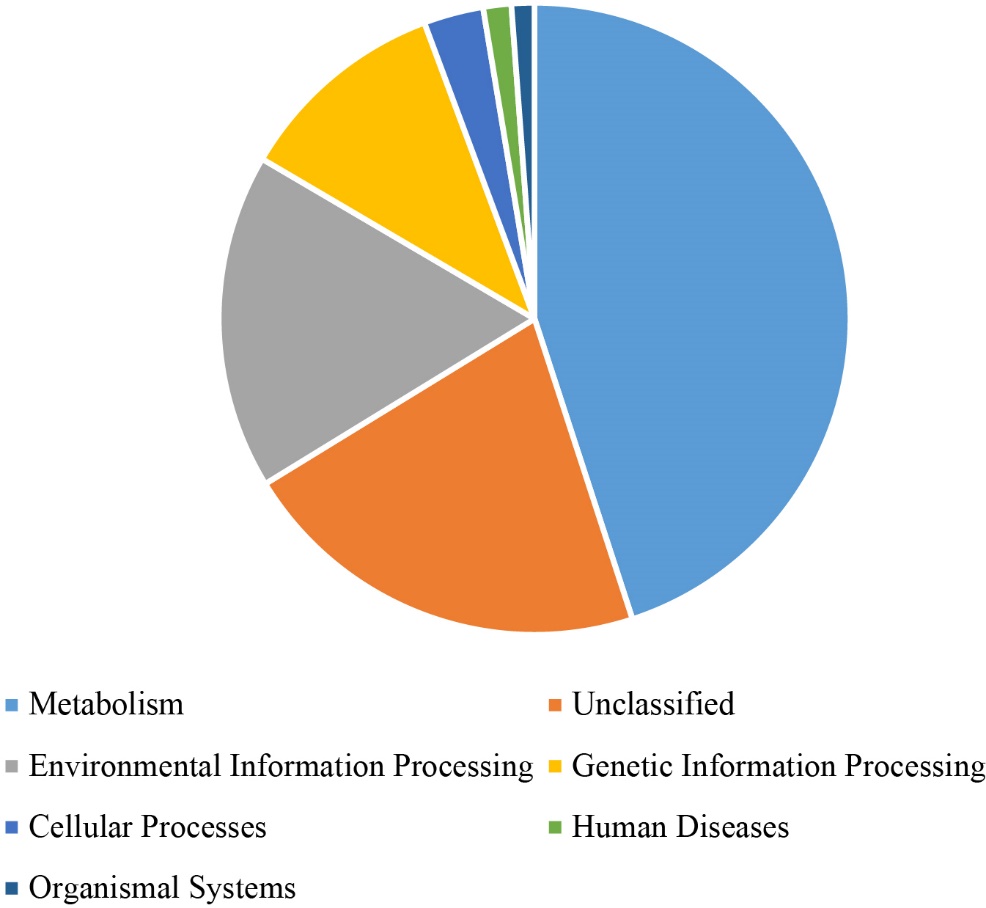


Fig S8 Profiles of different bacteria between goose and chicken fecal samples in KEGG class A


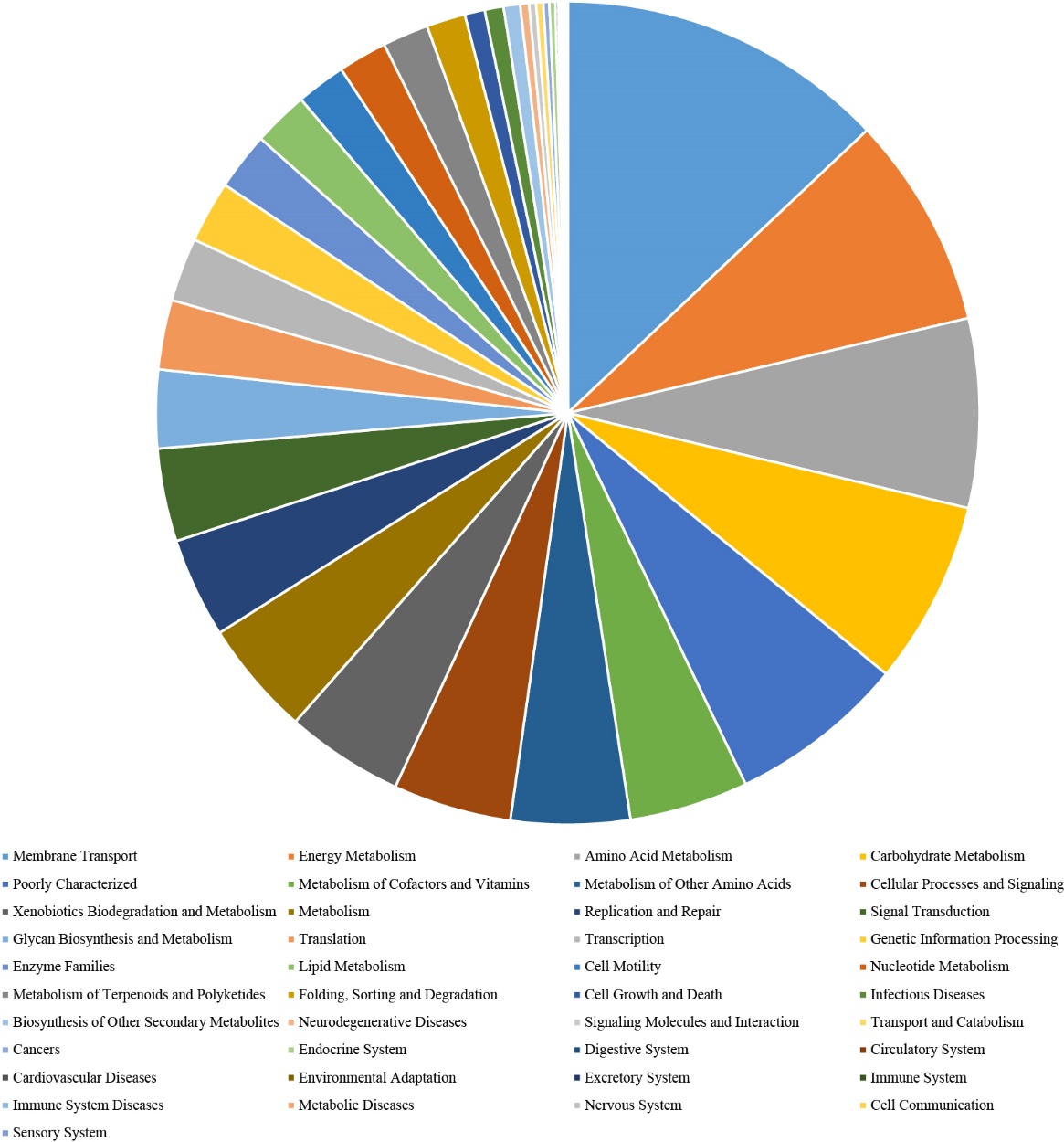


Fig S9 Profiles of different bacteria between goose and chicken fecal samples in KEGG class B

Supplementary Tables

Table S1 The depth and coverage of domestic and wild geese

| Samples | Coverage at least 1×(%) | Coverage at least 4×(%) | Coverage at least 20×(%) |
| --- | --- | --- | --- |
| Domestic goose | 99.92 | 99.50 | 91.13 |
| Wild goose | 99.68 | 99.07 | 88.40 |

Table S2 Strategy and statistics for the clean data of domestic goose genome sequncing

| Samples | Library | Reads  Num. | Total Bases  (bp) | Q20  (%) | Q30  (%) | GC  content |
| --- | --- | --- | --- | --- | --- | --- |
| Domestic Goose | PE400_1 | 79,700,468 | 17,498,494,689 | 94.9 | 93.06 | 43.39 |
|  | PE400_2 | 98,183,566 | 21,996,068,142 | 96.46 | 94.69 | 42.98 |
|  | PE700 | 45,889,474 | 9,957,618,364 | 96.08 | 92.38 | 42.11 |
|  | PE700_2 | 6,763,400 | 1,621,408,673 | 98.35 | 97.64 | 43.82 |
|  | MP2K | 27,878,946 | 5,218,508,812 | 97.29 | 90.71 | 46.17 |
|  | MP5K | 41,147,088 | 8,032,142,065 | 96.63 | 92.01 | 45.2 |
|  | MP10K | 13,167,360 | 3,015,226,956 | 99.05 | 95.94 | 44.91 |
|  | Total | 312,730,302 | 67,339,467,701 | 98.18 | 95.82 | 43.58 |
| Wild goose | PE400 | 473,803,082 | 57,849,313,902 | 98.18 | 95.82 | 45.29 |

Table S3 The size of domestic goose were estimated by the data of PE library

| Property | Value |
| --- | --- |
| K-mer | 17 |
| K-mer number | 1,510,890,418 |
| K-mer depth (×) | 39 |
| Low frequency k-mer number (≤2) | 622,023,873 |
| Average read length (bp) | 221,5 |
| Total reads number | 230.536,908 |
| Genome size (bp) | 1,198,802.839 |

Table S4 The annotated information of domestic goose genome

| Property | Value |
| --- | --- |
| Total Genes length | 509,444,683 |
| Genes Percentage of genome | 45.06% |
| Total Genes Number | 16,288 |
| Average gene length | 31,277 |
| Total Exons Number | 171,257 |
| Average Exons Per Gene | 10.5 |
| Total Exons length | 39,667,419 |
| Exons Percentage Of genome | 3.51% |
| Average Exons length | 231.6 |
| Average Introns length | 3,031 |
| Total CDSs length | 26,157,477 |
| CDSs Percentage of genome | 2.31% |
| Average CDS length | 1,606 |

Table S5 The repeat elements of domestic goose genome

| Elements | Number | Length (bp) | Percentage of Genome |
| --- | --- | --- | --- |
| Interspersed repeats | 190,289 | 63,216,951 | 5.7425% |
| Satellites | 2,503 | 288,004 | 0.0262% |
| Simple repeats | 7,648 | 1,091,578 | 0.0992% |
| Low complexity | 452 | 69,902 | 0.0063% |
| Summary | 284,441 | 76,007,230 | 6.9044% |

Table S6 Statistics of InDel in domestic goose genome

| Samples | Homozygous InDel | | Heterozygous InDels | | Total InDel | |
| --- | --- | --- | --- | --- | --- | --- |
|  | Number | Density | Number | Density | Number | Density |
| Domestic goose | 8,598 | 131,503 | 352,912 | 3,203 | 361,510 | 3,127 |
| Wild goose | 281,818 | 4,012 | 407,732 | 2,773 | 689,550 | 1,639 |

Table S7 Statistics of non-coding RNA in domestic goose genome

| ncRNA Type | Copy | Average length (bp) | Total length (bp) | % of genome |
| --- | --- | --- | --- | --- |
| rRNA | 12 | 498.08 | 5,977 | 0.00052% |
| tRNA | 204 | 75.65 | 15,434 | 0.00136% |
| snoRNA | 223 | 112.01 | 24,979 | 0.00227% |
| CD-box | 126 | 87.23 | 10,992 | 0.00097% |
| HACA-box | 81 | 138.65 | 11,231 | 0.00099% |
| scaRNA | 16 | 172.25 | 2,756 | 0.00024% |
| snRNA | 54 | 124.37 | 6,716 | 0.00061% |
| Other ncRNA | 345 | 106.26 | 36,659 | 0.00333% |
| Summary | 838 | 107.12 | 89,765 | 0.00815% |

Table S8. Statistics of function annotation of domestic goose genome

|  | Number | Percent (%) |
| --- | --- | --- |
| KEGG | 4503 | 27.65% |
| GO | 8704 | 53.44% |
| NR | 12085 | 74.20% |
| eggNOG | 11694 | 71.80% |
| At least in one database | 13541 | 83.13% |
| Unannotated | 2747 | 16.87% |
| Total | 16288 | 100.00% |

Table S9 Expanded and rapid evolving genes of goose genome involved in metabolism

| KO | Symbol | Description |
| --- | --- | --- |
| K00016 | LDH | L-lactate dehydrogenase |
| K01988 | A4GALT | lactosylceramide 4-alpha-galactosyltransferase |
| K00825 | AADAT | kynurenine/2-aminoadipate aminotransferase |
| K07513 | ACAA1 | acetyl-CoA acyltransferase 1 |
| K00255 | ACADL | long-chain-acyl-CoA dehydrogenase |
| K09478 | ACADSB | short/branched chain acyl-CoA dehydrogenase |
| K03392 | ACMSD | aminocarboxymuconate-semialdehyde decarboxylase |
| K01681 | ACO | aconitate hydratase |
| K11992 | ACOT8 | acyl-CoA thioesterase 8 |
| K01488 | add | adenosine deaminase |
| K00939 | adk | adenylate kinase |
| K01432 | AFMID | arylformamidase |
| K01196 | AGL | glycogen debranching enzyme |
| K01623 | ALDO | fructose-bisphosphate aldolase, class I |
| K03842 | ALG1 | beta-1,4-mannosyltransferase |
| K03848 | ALG6 | alpha-1,3-glucosyltransferase |
| K01490 | AMPD | AMP deaminase |
| K11182 | AOC1 | diamine oxidase |
| K00276 | AOC3 | primary-amine oxidase |
| K01940 | argG | argininosuccinate synthase |
| K02132 | ATPeF1A | F-type H+-transporting ATPase subunit alpha |
| K02147 | ATPeV1B | V-type H+-transporting ATPase subunit B |
| K03877 | B3GALT5 | beta-1,3-galactosyltransferase 5 |
| K00734 | B3GALT6 | galactosylxylosylprotein 3-beta-galactosyltransferase |
| K03766 | B3GNT5 | beta-1,3-N-acetylglucosaminyltransferase 5 |
| K00167 | BCKDHB | 2-oxoisovalerate dehydrogenase E1 component beta subunit |
| K00544 | BHMT | betaine-homocysteine S-methyltransferase |
| K12298 | CEL | bile salt-stimulated lipase |
| K07422 | CYP2U1 | cytochrome P450, family 2, subfamily U, polypeptide 1 |
| K04712 | DEGS | sphingolipid delta-4 desaturase |
| K09828 | DHCR24 | delta24-sterol reductase |
| K00254 | DHODH | dihydroorotate dehydrogenase |
| K00558 | DNMT1 | DNA (cytosine-5)-methyltransferase 1 |
| K00721 | DPM1 | dolichol-phosphate mannosyltransferase |
| K01464 | DPYS | dihydropyrimidinase |
| K01520 | dut | dUTP pyrophosphatase |
| K00472 | E1.14.11.2 | prolyl 4-hydroxylase |
| K07750 | E1.14.13.72 | methylsterol monooxygenase |
| K00643 | E2.3.1.37 | 5-aminolevulinate synthase |
| K00626 | E2.3.1.9 | acetyl-CoA C-acetyltransferase |
| K00772 | E2.4.2.28 | 5'-methylthioadenosine phosphorylase |
| K00826 | E2.6.1.42 | branched-chain amino acid aminotransferase |
| K00863 | E2.7.1.29 | dihydroxyacetone kinase |
| K01046 | E3.1.1.3 | triacylglycerol lipase |
| K01077 | E3.1.3.1 | alkaline phosphatase |
| K01092 | E3.1.3.25 | myo-inositol-1(or 4)-monophosphatase |
| K01099 | E3.1.3.36 | phosphatidylinositol-bisphosphatase |
| K01106 | E3.1.3.56 | inositol-1,4,5-trisphosphate 5-phosphatase |
| K01251 | E3.3.1.1 | adenosylhomocysteinase |
| K01583 | E4.1.1.19 | arginine decarboxylase |
| K01710 | E4.2.1.46 | dTDP-glucose 4,6-dehydratase |
| K01796 | E5.1.99.4 | alpha-methylacyl-CoA racemase |
| K14163 | EPRS | bifunctional glutamyl/prolyl-tRNA synthetase |
| K00993 | EPT1 | ethanolaminephosphotransferase |
| K02370 | EXTL3 | alpha-1,4-N-acetylglucosaminyltransferase EXTL3 |
| K00717 | FUT8 | glycoprotein 6-alpha-L-fucosyltransferase |
| K01084 | G6PC | glucose-6-phosphatase |
| K01132 | GALNS | N-acetylgalactosamine-6-sulfatase |
| K00710 | GALNT | polypeptide N-acetylgalactosaminyltransferase |
| K00613 | GATM | glycine amidinotransferase |
| K01201 | GBA | glucosylceramidase |
| K17108 | GBA2 | non-lysosomal glucosylceramidase |
| K02437 | gcvH | glycine cleavage system H protein |
| K00864 | glpK | glycerol kinase |
| K00600 | glyA | glycine hydroxymethyltransferase |
| K12409 | GNE | bifunctional UDP-N-acetylglucosamine 2-epimerase / N-acetylmannosamine kinase |
| K14455 | GOT2 | aspartate aminotransferase, mitochondrial |
| K00629 | GPAT1_2 | glycerol-3-phosphate O-acyltransferase 1/2 |
| K00049 | GRHPR | glyoxylate/hydroxypyruvate reductase |
| K00022 | HADH | 3-hydroxyacyl-CoA dehydrogenase |
| K05605 | HIBCH | 3-hydroxyisobutyryl-CoA hydrolase |
| K00760 | hprT | hypoxanthine phosphoribosyltransferase |
| K07965 | HPSE2 | heparanase 2 |
| K13368 | HSD17B2 | 17beta-estradiol 17-dehydrogenase / 3alpha(17beta)-hydroxysteroid dehydrogenase (NAD+) |
| K12405 | HSD17B4 | 3-hydroxyacyl-CoA dehydrogenase / 3a,7a,12a-trihydroxy-5b-cholest-24-enoyl-CoA hydratase |
| K13373 | HSD17B7 | 17beta-estradiol 17-dehydrogenase / 3beta-hydroxysteroid 3-dehydrogenase |
| K01197 | hya | hyaluronoglucosaminidase |
| K00030 | IDH3 | isocitrate dehydrogenase (NAD+) |
| K03334 | IL4I1 | L-amino-acid oxidase |
| K15759 | IMPAD1 | inositol monophosphatase 3 |
| K00253 | IVD | isovaleryl-CoA dehydrogenase |
| K01556 | KYNU | kynureninase |
| K10105 | LIPT1 | lipoyltransferase 1 |
| K01900 | LSC2 | succinyl-CoA synthetase beta subunit |
| K07512 | MECR | mitochondrial trans-2-enoyl-CoA reductase |
| K00789 | metK | S-adenosylmethionine synthetase |
| K00791 | miaA | tRNA dimethylallyltransferase |
| K01578 | MLYCD | malonyl-CoA decarboxylase |
| K13403 | MTHFD2 | methylenetetrahydrofolate dehydrogenase(NAD+) / 5,10-methenyltetrahydrofolate cyclohydrolase |
| K11352 | NDUFA12 | NADH dehydrogenase (ubiquinone) 1 alpha subcomplex subunit 12 |
| K03949 | NDUFA5 | NADH dehydrogenase (ubiquinone) 1 alpha subcomplex subunit 5 |
| K06210 | NMNAT | nicotinamide mononucleotide adenylyltransferase |
| K12669 | OST3 | oligosaccharyltransferase complex subunit gamma |
| K01966 | PCCB | propionyl-CoA carboxylase beta chain |
| K00967 | PCYT2 | ethanolamine-phosphate cytidylyltransferase |
| K00868 | pdxK | pyridoxine kinase |
| K00995 | pgsA | CDP-diacylglycerol--glycerol-3-phosphate 3-phosphatidyltransferase |
| K00888 | PI4K | phosphatidylinositol 4-kinase |
| K03860 | PIGQ | phosphatidylinositol glycan, class Q |
| K05292 | PIGT | phosphatidylinositol glycan, class T |
| K00763 | pncB | nicotinate phosphoribosyltransferase |
| K02325 | POLE2 | DNA polymerase epsilon subunit 2 |
| K02685 | PRI2 | DNA primase large subunit |
| K00318 | PRODH | proline dehydrogenase |
| K01613 | psd | phosphatidylserine decarboxylase |
| K08729 | PTDSS1 | phosphatidylserine synthase 1 |
| K15729 | PTGES | microsomal prostaglandin-E synthase 1 |
| K03783 | punA | purine-nucleoside phosphorylase |
| K00415 | QCR2 | ubiquinol-cytochrome c reductase core subunit 2 |
| K00357 | QDPR | dihydropteridine reductase |
| K11150 | RDH8 | retinol dehydrogenase 8 |
| K03012 | RPB4 | DNA-directed RNA polymerase II subunit RPB4 |
| K01807 | rpiA | ribose 5-phosphate isomerase A |
| K00314 | SARDH | sarcosine dehydrogenase |
| K08764 | SCP2 | sterol carrier protein 2 |
| K01565 | SGSH | N-sulfoglucosamine sulfohydrolase |
| K03369 | SIAT8E | alpha-N-acetyl-neuraminate alpha-2,8-sialyltransferase (sialyltransferase 8E) |
| K03370 | SIAT9 | lactosylceramide alpha-2,3-sialyltransferase (sialyltransferase 9) |
| K00797 | speE | spermidine synthase |
| K00654 | SPT | serine palmitoyltransferase |
| K00857 | tdk | thymidine kinase |
| K02377 | TSTA3 | GDP-L-fucose synthase |
| K00505 | TYR | tyrosinase |
| K00506 | TYRP1 | tyrosinase-related protein 1 |
| K00876 | udk | uridine kinase |
| K00699 | UGT | glucuronosyltransferase |
| K01195 | uidA | beta-glucuronidase |

Table S10. Profile of pathway in goose genome associate with reabsorb metabolites.

| Pathway | Cell | membre gene |  |
| --- | --- | --- | --- |
| insulin secretion (KO04911) | pancreatic beta cell | ATPase | Na k |
| Thyroid hormone synthesis (KO04918) | thyroid follicular cell | ATPase | Na k |
|  |  | NIS |  |
| pancreatic secretion（ko04972） | panceatic acinar cell | ATP | Na k |
|  |  | AE2 | H HCO3 |
|  | panceatic dust cell | ATP | Na k |
|  |  | NBCL | Na 2HCO3 |
|  |  | CFTR |  |
|  |  | SR CFTR | Cl |
| salivary secretion（ko04970） | salivary acinar cell | ATP | Na k |
|  |  | AE2 | HCO3 Cl |
|  |  | AC |  |
| gastric acid secretion（ko04971） | Gastric parietal cell | AC |  |
|  |  | AE | HCO3 Cl |
|  |  | Na/k | Na k |
|  |  | k/K | H CL K |
|  |  | CFTR |  |

Table S11. Statistics of SNPs heterozygote rates of coding and noncoding regions in wild and domestic goose

| Property | Domestic goose | Wild goose |
| --- | --- | --- |
| Genome | 0.002667737 | 0.004137166 |
| Gene | 0.002718533 | 0.004259656 |
| Gene interval | 0.002623981 | 0.004031653 |
| Intron | 0.002727 | 0.004263875 |
| Exon | 0.001930073 | 0.00306748 |
| Synonymous | 0.001055683 | 0.001648171 |
| Nonsynonymous | 0.000554564 | 0.000858034 |

Table S12 Summary the reads of fecal samples in goose and chicken

| Samples | Reads number | Samples | Reads number |
| --- | --- | --- | --- |
| Goose fecal 1 | 20,593 | Chicken fecal 1 | 18,233 |
| Goose fecal 2 | 57,784 | Chicken fecal 2 | 18,576 |
| Goose fecal 3 | 28,585 | Chicken fecal 3 | 26,736 |
| Goose fecal 4 | 69,649 | Chicken fecal 4 | 37,745 |
| Goose fecal 5 | 29,815 | Chicken fecal 5 | 17,280 |
| Goose fecal 6 | 20,502 | Chicken fecal 6 | 25,099 |
| Goose fecal 7 | 38,828 | Chicken fecal 7 | 27,241 |
| Goose fecal 8 | 22,988 | Chicken fecal 8 | 37,100 |
| Goose fecal 9 | 29,225 | Chicken fecal 9 | 53,591 |
| Goose fecal 10 | 20,024 | Chicken fecal 10 | 26,895 |
| Goose fecal 11 | 78,113 | Chicken fecal 11 | 22,646 |
| Goose fecal 12 | 23,899 | Chicken fecal 12 | 14,907 |
| Goose fecal 13 | 34,410 | Chicken fecal 13 | 42,954 |
| Goose fecal 14 | 14,608 | Chicken fecal 14 | 43,598 |
| Goose fecal 15 | 34,372 | Chicken fecal 15 | 33,397 |
| Goose fecal 16 | 22,877 | Chicken fecal 16 | 25,425 |
| Goose fecal 17 | 30,470 | Chicken fecal 17 | 24,081 |
| Goose fecal 18 | 26,240 | Chicken fecal 18 | 28,739 |
| Goose fecal 19 | 19,845 | Chicken fecal 19 | 54,180 |
| Goose fecal 20 | 23,107 | Chicken fecal 20 | 25,119 |
| Goose fecal 21 | 76,531 | Chicken fecal 21 | 26,488 |
| Goose fecal 22 | 9,851 | Chicken fecal 22 | 45,658 |
| Goose fecal 23 | 18,929 | Chicken fecal 23 | 32,949 |
| Goose fecal 24 | 23,616 | Chicken fecal 24 | 25,772 |
| Goose fecal 25 | 42,065 | Chicken fecal 25 | 26,972 |
| Goose fecal 26 | 29,565 | Chicken fecal 26 | 32,159 |
| Total of goose fecal | 846,491 | Chicken fecal 27 | 18,325 |
|  |  | Chicken fecal 28 | 18,700 |
|  |  | Chicken fecal 29 | 28,982 |
|  |  | Chicken fecal 30 | 21,836 |
|  |  | Total of chicken fecal | 881,383 |

Table S18 Diversity index. Indexes of Chao and Ace showed the gut microbes’ richness of each sample while indexes of Simpson and Shannon showed the uniformity of structure of gut microbes.

| Samples | Chao | ACE | Simpson | Shannon |
| --- | --- | --- | --- | --- |
| Goose fecal 1 | 797.5789 | 799.5436 | 0.1881 | 2.9044 |
| Goose fecal 2 | 771.6111 | 760.0936 | 0.1472 | 3.3337 |
| Goose fecal 3 | 998.2292 | 978.9064 | 0.0749 | 3.5628 |
| Goose fecal 4 | 916.9048 | 855.6496 | 0.0360 | 4.6549 |
| Goose fecal 5 | 936.1579 | 870.5055 | 0.0313 | 4.7323 |
| Goose fecal 6 | 815.0106 | 832.9637 | 0.2408 | 2.4207 |
| Goose fecal 7 | 1040.3490 | 1065.1686 | 0.1869 | 2.6063 |
| Goose fecal 8 | 997.4884 | 945.9138 | 0.0589 | 4.1311 |
| Goose fecal 9 | 1127.8592 | 1157.1489 | 0.1616 | 3.0823 |
| Goose fecal 10 | 1145.6207 | 1152.6514 | 0.0687 | 4.2568 |
| Goose fecal 11 | 929.5773 | 1346.7245 | 0.2687 | 2.3791 |
| Goose fecal 12 | 789.2019 | 792.7385 | 0.2589 | 2.2613 |
| Goose fecal 13 | 778.1915 | 798.1211 | 0.2072 | 2.5055 |
| Goose fecal 14 | 805.4455 | 796.5019 | 0.1850 | 2.6233 |
| Goose fecal 15 | 1040.6726 | 1042.8519 | 0.1093 | 3.2974 |
| Goose fecal 16 | 1056.7355 | 1050.3046 | 0.0398 | 4.8558 |
| Goose fecal 17 | 819.5946 | 879.5566 | 0.2015 | 2.7126 |
| Goose fecal 18 | 1238.1944 | 1218.4766 | 0.2665 | 2.6622 |
| Goose fecal 19 | 772.1010 | 763.8850 | 0.2521 | 2.3788 |
| Goose fecal 20 | 868.0000 | 865.3307 | 0.2107 | 2.7843 |
| Goose fecal 21 | 805.2500 | 1045.5647 | 0.2859 | 2.0052 |
| Goose fecal 22 | 656.4021 | 681.8348 | 0.2529 | 2.3078 |
| Goose fecal 23 | 1039.0074 | 1070.8565 | 0.0283 | 4.8004 |
| Goose fecal 24 | 824.3182 | 796.2806 | 0.1176 | 3.5937 |
| Goose fecal 25 | 1140.1139 | 1065.4410 | 0.0847 | 3.7332 |
| Goose fecal 26 | 911.5385 | 880.8671 | 0.0784 | 3.7114 |
| Chicken fecal 1 | 863.3125 | 806.8326 | 0.1570 | 3.0679 |
| Chicken fecal 2 | 1216.7823 | 1239.7265 | 0.0932 | 3.3739 |
| Chicken fecal 3 | 1165.0000 | 1170.9463 | 0.1039 | 3.4078 |
| Chicken fecal 4 | 1324.4559 | 1306.6159 | 0.2023 | 3.2545 |
| Chicken fecal 5 | 1322.7362 | 1344.3533 | 0.0755 | 3.4651 |
| Chicken fecal 6 | 2089.6411 | 2097.7858 | 0.0305 | 5.0900 |
| Chicken fecal 7 | 1584.5410 | 1568.2100 | 0.0955 | 3.6767 |
| Chicken fecal 8 | 1038.1250 | 1245.9633 | 0.1306 | 3.0421 |
| Chicken fecal 9 | 1125.4457 | 1172.3476 | 0.0887 | 3.3482 |
| Chicken fecal 10 | 1307.7343 | 1581.9550 | 0.0682 | 4.1173 |
| Chicken fecal 11 | 883.7596 | 1067.4628 | 0.1870 | 2.6278 |
| Chicken fecal 12 | 1402.4214 | 1375.0300 | 0.1178 | 3.4773 |
| Chicken fecal 13 | 1387.3542 | 1686.6040 | 0.1157 | 3.2465 |
| Chicken fecal 14 | 1383.1895 | 1458.0994 | 0.1072 | 3.1300 |
| Chicken fecal 15 | 1472.5617 | 1479.2883 | 0.1590 | 2.8699 |
| Chicken fecal 16 | 946.0729 | 1309.6776 | 0.4289 | 1.9013 |
| Chicken fecal 17 | 771.5616 | 861.5445 | 0.1036 | 2.9907 |
| Chicken fecal 18 | 895.7091 | 886.0070 | 0.1787 | 2.5892 |
| Chicken fecal 19 | 936.4741 | 966.0635 | 0.0628 | 3.7136 |
| Chicken fecal 20 | 616.7945 | 787.2215 | 0.2155 | 2.3719 |
| Chicken fecal 21 | 1032.4416 | 1371.6594 | 0.3478 | 2.1129 |
| Chicken fecal 22 | 1205.1707 | 1262.0020 | 0.0688 | 3.6834 |
| Chicken fecal 23 | 986.0101 | 966.0875 | 0.1165 | 3.0951 |
| Chicken fecal 24 | 1073.0593 | 1418.1479 | 0.1358 | 2.9626 |
| Chicken fecal 25 | 609.2632 | 530.3531 | 0.1950 | 2.2361 |
| Chicken fecal 26 | 923.6386 | 1169.1954 | 0.0198 | 4.4674 |
| Chicken fecal 27 | 907.9744 | 943.4629 | 0.1220 | 2.9432 |
| Chicken fecal 28 | 934.1683 | 936.7298 | 0.3880 | 1.8747 |
| Chicken fecal 29 | 890.2480 | 905.9231 | 0.1451 | 2.8232 |
| Chicken fecal 30 | 922.6786 | 941.2757 | 0.0843 | 3.2667 |
| Goose | 923±29 | 942±33 | 0.16±0.02 | 3.24±0.17 |
| Chicken | 1107±56 | 1195±59 | 0.14±0.02 | 3.14±0.13 |
| p value | 0.0076 | 0.0007 | 0.6669 | 0.6351 |

Table S22. Basic diet and nutrient levels of the diet of goose and chicken

| Raw material | Content (%) | Nutritional index | Nutritional level |
| --- | --- | --- | --- |
| Corn | 65.00 | Metabolizable energy | 13.39 |
| Soybean meal | 23.00 | Crude protein | 18.00 |
| Cottonseed meal | 3.50 | Lysine | 0.85 |
| Corn gluten meal | 3.00 | Met+Cys | 0.60 |
| Grease | 2.00 | Ca/% | 0.80 |
| Limestone | 1.00 | P | 0.65 |
| CaHPO4 | 1.20 |  |  |
| NaCl | 0.30 |  |  |
| Premix | 1.00 |  |  |

Per kg premix ingredients: VA 5000 IU/kg; VD 31000 IU/kg; VE 10 IU/kg; VK 0.5 IU/kg; pantothenic acid 10 mg; Niacin 30 mg; VB1 1.8 mg; VB2 3.5 mg; VB6 3.5 mg; biotin 0.1 mg; folic acid 0.55 mg; choline 1.0 g; VB12 0.012 mg; Mn 80 mg; Zn 60 mg; Fe 80 mg; Cu 8 mg; Se 0.15 mg.
